# Supplementary material for: Plasma microRNA signatures predict prognosis in canine osteosarcoma patients
Source: PLoS One. 2024 Dec 31;19(12):e0311104. doi: 10.1371/journal.pone.0311104 (PMC11687810; doi:10.1371/journal.pone.0311104)
Supplement: S1 Table — (DOCX) [file pone.0311104.s001.docx]

**S1 Table.** **MiRNA assay names included on the canine miScript miRNome (QIAGEN).**

| cfa-miR-383 | cfa-miR-129 | cfa-miR-135a-5p | cfa-miR-632 | cfa-miR-219-3p | cfa-miR-494 |
| --- | --- | --- | --- | --- | --- |
| cfa-miR-208b | cfa-miR-874 | cfa-miR-449a | cfa-miR-451 | cfa-miR-9 | cfa-miR-187 |
| cfa-miR-26a | cfa-miR-761 | cfa-miR-1837 | cfa-miR-331 | cfa-miR-146a | cfa-miR-429 |
| cfa-miR-872 | cfa-miR-34c | cfa-miR-367 | cfa-miR-181b | cfa-miR-411 | cfa-miR-96 |
| cfa-miR-10a | cfa-miR-27b | cfa-miR-206 | cfa-miR-375 | cfa-miR-504 | cfa-miR-106b |
| cfa-miR-302a | cfa-miR-1835 | cfa-miR-204 | cfa-miR-193a | cfa-miR-194 | cfa-miR-181a |
| cfa-miR-454 | cfa-miR-378 | cfa-miR-544 | cfa-miR-203 | cfa-miR-432 | cfa-miR-628 |
| cfa-miR-330 | cfa-miR-25 | cfa-miR-380 | cfa-miR-218 | cfa-miR-676 | cfa-miR-590 |
| cfa-let-7e | cfa-miR-124 | cfa-miR-496 | cfa-miR-10b | cfa-miR-433 | cfa-miR-885 |
| cfa-miR-211 | cfa-miR-384 | cfa-miR-33a | cfa-miR-342 | cfa-miR-217 | cfa-miR-1844 |
| cfa-miR-409 | cfa-miR-224 | cfa-miR-18b | cfa-miR-16 | cfa-miR-543 | cfa-miR-98 |
| cfa-miR-216b | cfa-miR-539 | cfa-miR-193b | cfa-miR-145 | cfa-miR-103 | cfa-miR-138a |
| cfa-miR-483 | cfa-miR-450b | cfa-miR-139 | cfa-miR-764 | cfa-miR-150 | cfa-miR-302b |
| cfa-miR-30c | cfa-miR-876 | cfa-miR-95 | cfa-miR-568 | cfa-miR-365 | cfa-miR-582 |
| cfa-miR-545 | cfa-miR-421 | cfa-miR-379 | cfa-miR-1306 | cfa-miR-127 | cfa-miR-190a |
| cfa-miR-27a | cfa-miR-28 | cfa-miR-490 | cfa-let-7b | cfa-miR-128 | cfa-miR-137 |
| cfa-miR-20b | cfa-miR-31 | cfa-miR-138b | cfa-miR-335 | cfa-miR-376a | cfa-miR-29a |
| cfa-miR-133b | cfa-miR-144 | cfa-miR-759 | cfa-miR-500 | cfa-miR-181c | cfa-miR-424 |
| cfa-miR-223 | cfa-miR-300 | cfa-miR-329b | cfa-miR-222 | cfa-miR-34a | cfa-miR-514 |
| cfa-miR-301a | cfa-miR-1 | cfa-miR-1307 | cfa-miR-152 | cfa-miR-325 | cfa-miR-122 |
| cfa-miR-197 | cfa-miR-30a | cfa-miR-551b | cfa-miR-299 | cfa-miR-1841 | cfa-miR-542 |
| cfa-let-7f | cfa-miR-105b | cfa-miR-1838 | cfa-miR-503 | cfa-miR-363 | cfa-miR-191 |
| cfa-miR-410 | cfa-miR-664 | cfa-miR-497 | cfa-miR-133a | cfa-miR-495 | cfa-miR-487b |
| cfa-miR-17 | cfa-miR-205 | cfa-miR-589 | cfa-miR-599 | cfa-miR-212 | cfa-miR-487a |
| cfa-miR-425 | cfa-miR-505 | cfa-miR-125b | cfa-miR-30e | cfa-miR-219-5p | cfa-miR-148a |
| cfa-miR-660 | cfa-miR-182 | cfa-let-7c | cfa-miR-200c | cfa-miR-136 | cfa-miR-326 |
| cfa-miR-382 | cfa-miR-215 | cfa-miR-221 | cfa-miR-190b | cfa-miR-148b | cfa-miR-101 |
| cfa-miR-350 | cfa-let-7j | cfa-miR-15b | cfa-miR-155 | cfa-miR-199 | cfa-miR-423a |
| cfa-miR-450a | cfa-miR-301b | cfa-miR-578 | cfa-miR-23b | cfa-miR-652 | cfa-miR-19b |
| cfa-miR-574 | cfa-let-7g | cfa-miR-592 | cfa-miR-302d | cfa-miR-489 | cfa-miR-153 |
| cfa-miR-488 | cfa-miR-22 | cfa-miR-7 | cfa-miR-135a-3p | cfa-miR-376b | cfa-miR-671 |
| cfa-miR-192 | cfa-miR-532 | cfa-miR-377 | cfa-miR-135b | cfa-miR-126 | cfa-miR-653 |
| cfa-miR-374a | cfa-miR-210 | cfa-miR-1842 | cfa-miR-143 | cfa-miR-329a | cfa-miR-371 |
| cfa-miR-125a | cfa-miR-184 | cfa-miR-181d | cfa-miR-324 | cfa-miR-140 | cfa-miR-1836 |
| cfa-miR-32 | cfa-miR-499 | cfa-miR-455 | cfa-miR-15a | cfa-miR-106a | cfa-miR-1839 |
| cfa-miR-132 | cfa-miR-345 | cfa-miR-361 | cfa-miR-21 | cfa-miR-34b | cfa-miR-186 |
| cfa-miR-502 | cfa-miR-92b | cfa-miR-29b | cfa-miR-802 | cfa-miR-29c | cfa-miR-24 |
| cfa-miR-130a | cfa-miR-146b | cfa-miR-376c | cfa-miR-23a | cfa-miR-493 | cfa-miR-188 |
| cfa-miR-875 | cfa-miR-374b | cfa-miR-151 | cfa-miR-202 | cfa-miR-452 | cfa-miR-183 |
| cfa-miR-338 | cfa-miR-320 | cfa-miR-185 | cfa-miR-207 | cfa-miR-485 | cfa-miR-448 |
| cfa-miR-142 | cfa-miR-130b | cfa-miR-18a | cfa-miR-195 | cfa-miR-381 | cfa-miR-196a |
| cfa-miR-30d | cfa-miR-362 | cfa-miR-328 | cfa-miR-93 | cfa-miR-99b | cfa-miR-20a |
| cfa-miR-340 | cfa-miR-92a | cfa-miR-200b | cfa-miR-141 | cfa-miR-26b | cfa-miR-369 |
| cfa-miR-105a | cfa-miR-708 | cfa-miR-200a | cfa-miR-216a | cfa-miR-758 | cfa-miR-149 |
| cfa-miR-214 | cfa-miR-491 | cfa-miR-134 | cfa-miR-1271 | cfa-let-7a | cfa-miR-551a |
| cfa-miR-208a | cfa-miR-19a | cfa-miR-665 | cfa-miR-196b | cfa-miR-30b | cfa-miR-99a |
| cfa-miR-133c |  |  |  |  |  |
| cel-miR-39-3p | cel-miR-39-3p | SNORD61 | SNORD68 | SNORD72 | SNORD95 |
| SNORD96A | RNU6-6P | miRTC | miRTC | PPC | PPC |

On the PCR array, there are 95 empty wells between last canine miRNA (cfa-miR-133c) and the 12 control wells.
