# Supplementary material for: Plasma microRNA signatures predict prognosis in canine osteosarcoma patients
Source: PLoS One. 2024 Dec 31;19(12):e0311104. doi: 10.1371/journal.pone.0311104 (PMC11687810; doi:10.1371/journal.pone.0311104)
Supplement: S2 Table — (DOCX) [file pone.0311104.s002.docx]

**S2 Table.** **MiRNA assay names and associated QIAGEN catalog numbers analyzed on the custom arrays.**

| **miRNA Assay Name** | **QIAGEN Catalog Number** | **miRNA Assay Name** | **QIAGEN Catalog Number** |
| --- | --- | --- | --- |
| bta-miR-195 | YP00205969 | hsa-miR-143-5p | YP00205992 |
| bta-miR-20b | YP00205943 | hsa-miR-145-5p | YP00204483 |
| bta-miR-26b | YP00205953 | hsa-miR-148b-3p | YP00204047 |
| cel-miR-39-3p | YP00203952 | hsa-miR-151a-5p | YP00204007 |
| cfa-miR-1 | YP02119135 | hsa-miR-16-5p | YP00205702 |
| cfa-miR-125a | YP02113289 | hsa-miR-185-5p | YP00206037 |
| cfa-miR-1271 | YP02112462 | hsa-miR-19a-3p | YP00205862 |
| cfa-miR-1306 | YP02110639 | hsa-miR-205-5p | YP00204487 |
| cfa-miR-133c | YP02100855 | hsa-miR-20a-5p | YP00204292 |
| cfa-miR-138b | YP02117682 | hsa-miR-214-3p | YP00204510 |
| cfa-miR-140 | YP00205973 | hsa-miR-22-3p | YP00204606 |
| cfa-miR-142 | YP02102101 | hsa-miR-222-3p | YP00204551 |
| cfa-miR-144 | YP02107159 | hsa-miR-27b-3p | YP00205915 |
| cfa-miR-210 | YP02117670 | hsa-miR-28-3p | YP00204119 |
| cfa-miR-221 | YP02104713 | hsa-miR-378a-3p | YP00205946 |
| cfa-miR-23a | YP00205956 | hsa-miR-433-3p | YP00204036 |
| cfa-miR-23b | YP00205959 | hsa-miR-451a | YP02119305 |
| cfa-miR-30a | YP02101182 | hsa-miR-505-5p | YP00205657 |
| cfa-miR-589 | YP02109686 | hsa-miR-551a | YP00204331 |
| cfa-miR-652 | YP02104033 | hsa-miR-7-5p | YP00205877 |
| dme-miR-133-3p | YP00205954 | hsa-miR-802 | YP00205980 |
| gga-miR-18a-5p | YP02100185 | hsa-miR-885-5p | YP00204473 |
| hsa-let-7c-5p | YP00204767 | hsa-miR-92a-3p | YP00204258 |
| hsa-miR-125b-5p | YP00205713 | hsa-miR-92b-3p | YP00204384 |
| hsa-miR-126-5p | YP00206010 | hsa-miR-93-5p | YP00204715 |
| hsa-miR-128-3p | YP00205995 | rno-miR-223-3p | YP00205120 |
| hsa-miR-1307-3p | YP02103132 | UniSp3 | YP02119288 |
| hsa-miR-133b | YP00206058 | UniSp6 | YP00203954 |
