# Supplementary material for: Plasma microRNA signatures predict prognosis in canine osteosarcoma patients
Source: PLoS One. 2024 Dec 31;19(12):e0311104. doi: 10.1371/journal.pone.0311104 (PMC11687810; doi:10.1371/journal.pone.0311104)
Supplement: S3 Table — (DOCX) [file pone.0311104.s003.docx]

**S3 Table. Demographics of the dogs included in each population group.**

|  | **OVC1 (n = 35)** | **OVC2 (n = 13)** | **CCOGC (n = 13)** | **Controls (n = 21)** |
| --- | --- | --- | --- | --- |
| **Breeds** | Mixed breed: 12  Saint Bernard: 2  Golden Retriever: 2  Great Dane: 2  Greyhound: 2  Boxer: 2  Labrador Retriever: 1  Australian Shepherd: 1  Great Pyrenees: 1  Bernese Mountain Dog: 1  Standard Poodle: 1  English Bulldog: 1  Rottweiler: 1  Dogue de Bordeaux: 1  Akita: 1  Catahoula Leopard Dog: 1  Newfoundland: 1  Pyrenean Mastiff: 1  German Shepherd: 1 | Rottweiler: 3  Golden Retriever: 3  Doberman: 1  Australian Shepherd: 1  Standard Poodle: 1  Labrador Retriever: 1  Mixed breed: 1  Great Pyrenees: 1  Greyhound: 1 | Mixed breed: 4  Greyhound: 3  Great Dane: 1  Labrador Retriever: 1  Leonberger: 1  Old English Sheepdog: 1  Belgian Tervuren: 1  Golden Retriever: 1 | Mixed breed: 6  Labrador Retriever: 4  Bull Mastiff: 2  Bernese Mountain Dog: 2  Border Collie: 1  Standard Poodle: 1  Flat-Coated Retriever: 1  English Bulldog: 1  German Shorthaired Pointer: 1  Newfoundland: 1  Cockapoo: 1 |
| **Location of tumour** | Distal radius: 15  Proximal humerus: 7  Distal femur: 4  Distal tibia: 4  Proximal tibia: 3  Proximal ulna: 1  Scapula: 1 | Proximal humerus: 3  Distal radius: 3  Proximal tibia: 3  Distal tibia: 2  Proximal femur: 1  Distal femur: 1 | Femur: 5  Radius: 3  Humerus: 3  Tibia: 1  Ulna: 1 | N/A |
| **Sex** | Spayed female: 17  Neutered male: 17  Intact male: 1  Intact female: 0 | Spayed female: 5  Neutered male: 5  Intact female: 2  Intact male: 1 | Neutered male: 7  Spayed female: 6  Intact male: 0  Intact female: 0 | Neutered male: 9  Spayed female: 8  Intact female: 3  Intact male: 1 |
| **Age (years)** | Median: 8.48  Range: 2.66-12.07 | Median: 8.28  Range: 5.47-12.44 | Median: 7  Range: 5-11 | Median: 4.58  Range: 1.22-10.00 |
| **Weight (kg)** | Median: 38.50  Range: 23.8-69.0 | Median: 33.20  Range: 22.0-63.0 | Median: 33.4  Range: 21.0-56.4 | *Median: 35.1  *Range: 19.5-59.0 |
| **Institution** | OVC | OVC | OSU: 6  Wisconsin: 4  C.L. Davis: 2  Tufts: 1 | OVC |

*Weight was not available for four control dogs.
