# Supplementary material for: Plasma microRNA signatures predict prognosis in canine osteosarcoma patients
Source: PLoS One. 2024 Dec 31;19(12):e0311104. doi: 10.1371/journal.pone.0311104 (PMC11687810; doi:10.1371/journal.pone.0311104)
Supplement: S4 Table — (DOCX) [file pone.0311104.s004.docx]

**S4 Table. Clinical outcome parameters for each OSA population.**

| **Population** | **Overall survival (median days)** | **Disease-free interval (median days)** | **1-year survival**  **(percent alive)** |
| --- | --- | --- | --- |
| **OVC1** | 277 | 260 | 42.86% |
| **OVC2** | 344 | 244 | 38.46% |
| **CCOGC** | 754 | 610* | 61.54% |

*Only 12/13 dogs were considered in this calculation due to reliability of clinical information.
