# Supplementary material for: Plasma microRNA signatures predict prognosis in canine osteosarcoma patients
Source: PLoS One. 2024 Dec 31;19(12):e0311104. doi: 10.1371/journal.pone.0311104 (PMC11687810; doi:10.1371/journal.pone.0311104)
Supplement: S5 Table — (DOCX) [file pone.0311104.s005.docx]

**S5 Table. MiRNAs differentially expressed between OSA populations and healthy controls.**

|  | **OVC1** | | | **OVC2** | | | **CCOGC** | | |
| --- | --- | --- | --- | --- | --- | --- | --- | --- | --- |
| **miRNA** | **Median**  **fold-difference** | **p-value** | **FDR adjusted p-value** | **Median**  **fold-difference** | **p-value** | **FDR adjusted p-value** | **Median fold-difference** | **p-value** | **FDR adjusted p-value** |
| bta-miR-195 | 1.42 | 6.73E-02 | 1.74E-01 | **1.82** | **1.97E-02** | **3.94E-02** | 1.56 | 1.58E-01 | 3.03E-01 |
| bta-miR-20b | -1.20 | 2.25E-01 | 3.81E-01 | ***-5.26*** | ***2.09E-07*** | ***1.84E-06*** | 1.92 | 7.03E-02 | 1.55E-01 |
| bta-miR-26b | -1.31 | 6.23E-02 | 1.71E-01 | **-1.48** | **1.60E-02** | **3.71E-02** | -1.56 | 2.18E-01 | 3.69E-01 |
| cfa-miR-1 | ***2.98*** | ***1.08E-02*** | ***4.17E-02*** | 2.20 | 8.91E-02 | 1.45E-01 | 5.40 | 2.41E-02 | 6.23E-02 |
| cfa-miR-125a | **1.75** | **1.14E-02** | **4.17E.02** | -1.74 | 5.48E-02 | 1.01E-01 | ***3.46*** | ***1.41E-04*** | ***7.77E-04*** |
| cfa-miR-1271 | -1.05 | 6.26E-01 | 7.59E-01 | -1.86 | 1.69E-01 | 2.13E-01 | 1.56 | 5.52E-01 | 6.75E-01 |
| cfa-miR-133c | ***5.30*** | ***1.20E-04*** | ***1.06E-.03*** | 2.21 | 1.69E-01 | 2.13E-01 | ***9.80*** | ***2.58E-06*** | ***1.14E-04*** |
| cfa-miR-1306 | 2.02 | 3.21E-01 | 4.67E-01 | **-1.74** | **1.78E-02** | **3.91E-02** | 2.91 | 2.18E-02 | 5.99E-02 |
| cfa-miR-140 | 1.40 | 3.29E-01 | 4.67E-01 | -1.65 | 6.48E-02 | 1.14E-01 | 1.67 | 9.62E-02 | 2.01E-01 |
| cfa-miR-142 | -1.36 | 1.01E-01 | 2.34E-01 | -1.14 | 1.58E-01 | 2.11E-01 | 1.11 | 9.17E-01 | 9.38E-01 |
| cfa-miR-144 | 1.79 | 9.07E-01 | 9.07E-01 | ***-6.26*** | ***8.02E-05*** | ***2.52E-04*** | 1.16 | 5.76E-01 | 6.85E-01 |
| cfa-miR-221 | -1.40 | 1.24E-01 | 2.60E-01 | ***-18.66*** | ***2.59E-08*** | ***3.79E-07*** | 1.44 | 3.09E-01 | 4.38E-01 |
| cfa-miR-23a | 1.084 | 8.93E-01 | 9.07E-01 | -1.41 | 1.12E-01 | 1.75E-01 | 1.30 | 2.76E-01 | 4.34E-01 |
| cfa-miR-30a | ***2.86*** | ***1.37E-03*** | ***8.60E-03*** | -1.32 | 5.29E-01 | 5.82E-01 | ***3.66*** | ***4.00E-04*** | ***1.95E-03*** |
| cfa-miR-652 | 1.41 | 5.34E-01 | 6.72E-01 | ***-5.16*** | ***3.39E-06*** | ***1.86E-05*** | 1.88 | 5.03E-02 | 1.23E-01 |
| dme-miR-133-3p | ***4.24*** | ***4.11E-05*** | ***4.52E-04*** | 2.16 | 3.81E-01 | 4.41E-01 | ***9.47*** | ***2.90E-05*** | ***4.25E-04*** |
| gga-miR-18a-5p | -1.24 | 1.42E-01 | 2.71E-01 | ***-9.72*** | ***4.31E-09*** | ***1.90E-07*** | 1.64 | 5.29E-01 | 6.65E-01 |
| hsa-let-7c-5p | -1.44 | 5.76E-02 | 1.69E-01 | -1.83 | 7.03E-02 | 1.19E-01 | 1.11 | 7.27E-01 | 8.20E-01 |
| hsa-miR-125b-5p | ***2.13*** | ***8.34E-03*** | ***3.67E-02*** | **-1.49** | **1.97E-02** | **3.94E-02** | ***4.10*** | ***8.79E-04*** | ***3.87E-03*** |
| hsa-miR-126-5p | 1.01 | 2.59E-01 | 4.07E-01 | 1.75 | 1.20E-01 | 1.76E-01 | -1.24 | 1.29E-01 | 2.58E-01 |
| hsa-miR-128-3p | 1.27 | 4.60E-01 | 6.08E-01 | ***-4.15*** | ***1.47E-06*** | ***9.23E-06*** | 2.21 | 2.92E-01 | 4.38E-01 |
| hsa-miR-1307-3p | -1.11 | 8.27E-01 | 8.67E-01 | ***-4.99*** | ***3.58E-05*** | ***1.31E-04*** | **1.98** | **7.39E-03** | **2.32E-02** |
| hsa-miR-133b | ***6.48*** | ***1.28E-05*** | ***2.06E-04*** | 1.08 | 5.06E-01 | 5.71E-01 | ***9.38*** | ***9.72E-05*** | ***6.11E-04*** |
| hsa-miR-143-5p | ***2.49*** | ***5.12E-03*** | ***2.50E-02*** | 1.14 | 7.53E-01 | 8.08E-01 | ***5.50*** | ***9.72E-05*** | ***6.11E-04*** |
| hsa-miR-145-5p | ***3.77*** | ***1.40E-05*** | ***2.06E-04*** | 1.35 | 2.61E-01 | 3.10E-01 | ***3.85*** | ***5.40E-05*** | ***5.81E-04*** |
| hsa-miR-148b-3p | -1.33 | 2.81E-02 | 9.53E-02 | ***-4.48*** | ***6.47E-08*** | ***7.11E-07*** | 1.15 | 8.34E-01 | 9.17E-01 |
| hsa-miR-151a-5p | **-1.88** | **4.32E-03** | **2.38E-02** | -1.15 | 1.20E-01 | 1.76E-01 | -1.17 | 5.29E-01 | 6.65E-01 |
| hsa-miR-16-5p | 1.70 | 3.05E-01 | 4.62E-01 | 1.10 | 1.58E-01 | 2.11E-01 | 2.29 | 3.62E-01 | 4.83E-01 |
| hsa-miR-185-5p | -1.06 | 6.87E-01 | 7.96E-01 | ***-3.66*** | ***6.60E-05*** | ***2.23E-04*** | 2.34 | 1.93E-01 | 3.39E-01 |
| hsa-miR-19a-3p | -1.66 | 1.20E-01 | 2.60E-01 | ***-5.34*** | ***1.87E-05*** | ***7.47E-05*** | 1.66 | 2.76E-01 | 4.34E-01 |
| hsa-miR-205-5p | 2.63 | 5.53E-02 | 1.69E-01 | ***-2.79*** | ***1.18E-03*** | ***3.25E-03*** | 1.57 | 3.26E-01 | 4.48E-01 |
| hsa-miR-20a-5p | -1.28 | 1.56E-01 | 2.87E-01 | ***-9.03*** | ***2.59E-08*** | ***3.79E-07*** | 1.26 | 9.44E-01 | 9.44E-01 |
| hsa-miR-214-3p | ***5.16*** | ***1.39E-06*** | ***6.13E-05*** | **1.96** | **2.18E-02** | ***4.17E-02*** | ***8.87*** | ***7.30E-06*** | ***1.61E-04*** |
| hsa-miR-22-3p | 1.49 | 4.30E-01 | 5.91E-01 | ***-4.19*** | ***7.30E-06*** | ***3.57E-05*** | ***2.05*** | ***3.11E-03*** | ***1.14E-02*** |
| hsa-miR-222-3p | 1.38 | 4.70E-01 | 6.08E-01 | ***-2.80*** | ***1.16E-02*** | ***2.84E-02*** | 1.92 | 1.97E-02 | 5.78E-02 |
| hsa-miR-28-3p | -1.22 | 1.42E-01 | 2.71E-01 | 1.09 | 9.17E-01 | 9.38E-01 | 1.09 | 8.61E-01 | 9.24E-01 |
| hsa-miR-378a-3p | ***2.16*** | ***1.20E-03*** | ***8.60E-03*** | 1.35 | 1.93E-01 | 2.35E-01 | ***2.07*** | ***6.60E-05*** | ***5.81E-04*** |
| hsa-miR-451a | -1.54 | 2.45E-01 | 4.00E-01 | ***-6.30*** | ***9.31E-06*** | ***4.10E-05*** | 1.17 | 8.89E-01 | 9.31E-01 |
| hsa-miR-505-5p | 1.18 | 6.38E-01 | 7.59E-01 | -1.63 | 1.29E-01 | 1.83E-01 | 2.04 | 7.03E-02 | 1.55E-01 |
| hsa-miR-7-5p | 1.12 | 7.88E-01 | 8.46E-01 | ***-2.54*** | ***9.30E-03*** | ***2.41E-02*** | 1.78 | 6.24E-01 | 7.23E-01 |
| hsa-miR-885-5p | 2.01 | 8.23E-02 | 2.06E-01 | 1.38 | 9.44E-01 | 9.44E-01 | ***3.69*** | ***6.57E-03*** | ***2.22E-02*** |
| hsa-miR-92a-3p | 1.66 | 7.37E-01 | 8.25E-01 | ***-2.37*** | ***2.86E-04*** | ***8.39E-04*** | 2.97 | 1.81E-01 | 3.31E-01 |
| hsa-miR-92b-3p | 1.55 | 1.83E-01 | 3.22E-01 | 1.09 | 9.17E-01 | 9.38E-01 | ***3.12*** | ***1.02E-03*** | ***4.09E-03*** |
| hsa-miR-93-5p | -1.12 | 7.50E-01 | 8.25E-01 | ***-7.25*** | ***1.47E-06*** | ***9.23E-06*** | 2.02 | 3.09E-01 | 4.38E-01 |

MiRNAs with statistically significant fold-differences in bold (by FDR-adjusted p-value). Those with a fold-difference +/- 2 also italicized.
