# Supplementary material for: Plasma microRNA signatures predict prognosis in canine osteosarcoma patients
Source: PLoS One. 2024 Dec 31;19(12):e0311104. doi: 10.1371/journal.pone.0311104 (PMC11687810; doi:10.1371/journal.pone.0311104)
Supplement: S6 Table — (DOCX) [file pone.0311104.s006.docx]

**S6 Table. MiRNA associations with hemolysis.**

| **MiRNA** | **R^2^ of linear trend line** | **p-value of hemolyzed to non-hemolyzed group comparison (Mann Whitney U test)** |
| --- | --- | --- |
| **Strong association with hemolysis** | | |
| hsa-miR-16-5p | 0.41 | 0.00027 |
| hsa-miR-505-5p | 0.41 | 0.0029 |
| cfa-miR-1306 | 0.38 | 0.00071 |
| hsa-miR-451a | 0.37 | 0.00027 |
| hsa-miR-92a-3p | 0.35 | 0.0024 |
| gga-miR-18a-5p | 0.33 | 0.0091 |
| cfa-miR-1271 | 0.33 | 0.014 |
| hsa-miR-93-5p | 0.32 | 0.0067 |
| bta-miR-20b | 0.31 | 0.025 |
| hsa-miR-128-3p | 0.30 | 0.011 |
| cfa-miR-652 | 0.30 | 0.0096 |
| hsa-miR-7-5p | 0.30 | 0.0073 |
| **Poor association with hemolysis** | | |
| bta-miR-26b | 0.18 | 0.054 |
| hsa-miR-22-3p | 0.14 | 0.28 |
| hsa-miR-222-3p | 0.10 | 0.078 |
| cfa-miR-221 | 0.092 | 0.51 |
| hsa-miR-28-3p | 0.074 | 0.17 |
| dme-miR-133-3p | 0.074 | 0.39 |
| rno-miR-223-3p | 0.072 | 0.11 |
| cfa-miR-23a | 0.072 | 0.19 |
| cfa-miR-30a | 0.070 | 0.26 |
| hsa-miR-133b | 0.06 | 0.5 |
| hsa-miR-378a-3p | 0.052 | 0.13 |
| hsa-miR-27b-3p | 0.047 | 0.25 |
| hsa-miR-151a-5p | 0.046 | 0.33 |
| cfa-miR-133c | 0.045 | 0.63 |
| cfa-miR-23b | 0.038 | 0.86 |
| hsa-miR-885-5p | 0.036 | 0.6 |
| hsa-miR-126-5p | 0.035 | 0.4 |
| bta-miR-195 | 0.032 | 0.34 |
| cfa-miR-1 | 0.016 | 0.79 |
| hsa-miR-214-3p | 0.011 | 0.33 |
| hsa-miR-92b-3p | 0.002 | 0.9 |
| **Undetermined association with hemolysis** | | |
| hsa-miR-1307-3p | 0.28 | 0.12 |
| hsa-miR-20a-5p | 0.27 | 0.017 |
| hsa-miR-19a-3p | 0.27 | 0.0043 |
| hsa-miR-148b-3p | 0.27 | 0.064 |
| cfa-miR-142 | 0.26 | 0.011 |
| hsa-miR-185-5p | 0.25 | 0.03 |
| hsa-miR-125b-5p | 0.24 | 0.013 |
| cfa-miR-144 | 0.23 | 0.17 |
| cfa-miR-140 | 0.21 | 0.017 |
| hsa-let-7c-5p | 0.19 | 0.018 |
| cfa-miR-125a | 0.15 | 0.016 |
| hsa-miR-205-5p | 0.13 | 0.04 |
| hsa-miR-143-5p | 0.11 | 0.0032 |
| hsa-miR-145-5p | 0.07 | 0.0044 |
