# Supplementary material for: Plasma microRNA signatures predict prognosis in canine osteosarcoma patients
Source: PLoS One. 2024 Dec 31;19(12):e0311104. doi: 10.1371/journal.pone.0311104 (PMC11687810; doi:10.1371/journal.pone.0311104)
Supplement: S7 Table — (DOCX) [file pone.0311104.s007.docx]

**S7 Table. MiRNAs associated with disease-free interval in each population.**

| **OVC1** | | | | |
| --- | --- | --- | --- | --- |
| **miRNA** | **Cut-off value*** | **Median days in high group (sample #)** | **Median days in low group (sample #)** | **p-value** |
| **hsa.miR.214.3^a^** | 3.50 | 67 (15) | 364 (20) | 3.85E-05 |
| **cfa.miR.652** | 4.89 | 264 (27) | 68.5 (8) | 4.12E-05 |
| **gga.miR.18a.5p** | 4.78 | 264 (27) | 81 (8) | 4.43E-05 |
| **cfa.miR.23a** | -0.45 | 79 (11) | 265 (24) | 6.96E-04 |
| **hsa.miR.93.5p^b^** | 0.57 | 264 (25) | 73.5 (10) | 7.63E-04 |
| **hsa.miR.20a.5p^b^** | 0.23 | 266 (23) | 81 (12) | 1.42E-03 |
| **hsa.miR.378a.3p** | 4.24 | 106 (25) | 563.5 (10) | 4.65E-03 |
| **cfa.miR.1271** | 5.61 | 106 (13) | 263.5 (22) | 8.38E-03 |
| **bta.miR.20b^b^** | 4.36 | 265 (24) | 86 (11) | 1.10E-02 |
| **hsa.miR.185.5p^b^** | 4.99 | 264 (23) | 68.5 (12) | 1.38E-02 |
| **hsa.miR.205.5p** | 3.62 | 266 (17) | 117 (18) | 1.46E-02 |
| **hsa.miR.133b** | 3.74 | 264 (17) | 168.5 (18) | 1.73E-02 |
| **hsa.miR.451a** | -5.86 | 438 (11) | 117 (24) | 2.36E-02 |
| **hsa.miR.143.3p^b^** | 6.33 | 117 (22) | 273 (13) | 2.58E-02 |
| **hsa.miR.92a.3p** | -2.50 | 243.5 (24) | 128 (11) | 2.86E-02 |
| **cfa.miR.221^b^** | 2.63 | 266 (23) | 91.5 (12) | 3.14E-02 |
| **hsa.miR.28.3p^c^** | 3.82 | 79 (9) | 244 (26) | 3.49E-02 |
| **hsa.miR.222.3p** | 2.60 | 440.5 (8) | 139 (27) | 4.40E-02 |
| **hsa.miR.126.5p** | 3.60 | 107.5 (14) | 261 (21) | 4.56E-02 |
| **cfa.miR.142** | 1.82 | 260.5 (28) | 53 (7) | 4.78E-02 |
| **hsa.miR.22.3p^c^** | 3.03 | 265 (24) | 97 (11) | 4.87E-02 |
| **OVC2** | | | | |
| **miRNA** | **Cut-off value*** | **Median days in high group (sample #)** | **Median days in low group (sample #)** | **p-value** |
| **hsa.miR.214.3p^a^** | 4.27 | 92 (5) | 307.5 (8) | 6.38E-04 |
| **hsa.miR.19a.3p** | 1.25 | 391 (6) | 112(7) | 7.27E-04 |
| **hsa.miR.143.3p^b^** | 5.09 | 73 (2) | 244 (11) | 1.88E-03 |
| **cfa.miR.221^b^** | 6.74 | 271 (9) | 102 (4) | 2.64E-03 |
| **dme.miR.133.3p** | 4.39 | 92 (3) | 257.5 (10) | 4.45E-03 |
| **hsa.miR.22.3p** | 5.25 | 112 (9) | 574.5 (4) | 1.37E-02 |
| **hsa.miR.28.3p** | 4.20 | 354.5 (6) | 92 (7) | 1.72E-02 |
| **hsa.miR.93.5p^b^** | 3.31 | 257.5 (10) | 112 (3) | 2.57E-02 |
| **hsa.miR.20a.5p^b^** | 2.73 | 271 (9) | 115 (4) | 2.88E-02 |
| **hsa.miR.151a.5p** | 3.85 | 271 (7) | 92 (6) | 3.69E-02 |
| **cfa.miR.652** | 5.26 | 86 (2) | 244 (11) | 4.18E-02 |
| **hsa.miR.185.5p^b^** | 6.57 | 307.5 (8) | 118 (5) | 4.72E-02 |
| **bta.miR.20b^b^** | 6.13 | 271 (7) | 115 (6) | 4.91E-02 |
| **CCOGC** | | | | |
| **miRNA** | **Cut-off value*** | **Median days in high group (sample #)** | **Median days in low group (sample #)** | **p-value** |
| **hsa.miR.92b.3p** | 4.87 | 87.5 (2) | 682 (10) | 1.68E-02 |
| **hsa.miR.143.3p** | 3.14 | 1198 (2) | 290 (10) | 2.69E-02 |
| **hsa.miR.885.5p** | 4.42 | 1198 (2) | 290 (10) | 2.69E-02 |
| **hsa.let.7c.5p** | 0.91 | 97 (3) | 754 (9) | 3.39E-02 |
| **hsa.miR.28.3p^c^** | 3.48 | 97 (3) | 754 (9) | 3.39E-02 |
| **hsa.miR.22.3p^c^** | 1.74 | 819 (6) | 104.5 (6) | 3.76E-02 |
| **hsa.miR.214.3p^a^** | 2.27 | 97 (4) | 704.5 (8) | 4.71E-02 |
| **hsa.miR.378a.3p** | 2.27 | 1119 (3) | 112 (9) | 4.87E-02 |

^a^ miRNAs with the same disease-free interval groups in all three populations.

^b^ miRNAs with the same disease-free interval groups in OVC1 and OVC2.

^c^ miRNAs with the same disease-free interval groups in OVC1 and CCOGC.

*Cut-off value represents the normalized Ct value of the respective miRNA which best separates the high group and low group.

Note: No miRNA predicted disease-free interval similarly between OVC2 and CCOGC.
