# Supplementary material for: Plasma microRNA signatures predict prognosis in canine osteosarcoma patients
Source: PLoS One. 2024 Dec 31;19(12):e0311104. doi: 10.1371/journal.pone.0311104 (PMC11687810; doi:10.1371/journal.pone.0311104)
Supplement: S8 Table — (DOCX) [file pone.0311104.s008.docx]

**S8 Table. MiRNAs associated with overall survival in each population.**

| **OVC1** | | | | |
| --- | --- | --- | --- | --- |
| **miRNA** | **Cut-off value*** | **Median days in high group (sample #)** | **Median days in low group (sample #)** | **p-value** |
| **cfa.miR.652** | 4.89 | 295 (27) | 81 (8) | 3.36E-06 |
| **hsa.miR.214.3p^a^** | 3.50 | 92 (15) | 446 (20) | 1.79E-05 |
| **gga.miR.18a.5p^b^** | 4.78 | 295 (27) | 89 (8) | 7.14E-05 |
| **hsa.miR.93.5p^b^** | 0.57 | 338 (25) | 107.5 (10) | 4.06E-04 |
| **hsa.miR.20a.5p** | 0.23 | 338 (23) | 107.5 (12) | 1.34E-03 |
| **cfa.miR.23a** | -0.45 | 123 (11) | 316.5 (24) | 1.97E-03 |
| **hsa.miR.378a.3p** | 4.24 | 138 (25) | 690.5 (10) | 3.03E-03 |
| **cfa.miR.1271** | 5.61 | 138 (13) | 290.5 (22) | 8.14E-03 |
| **hsa.miR.205.5p** | 3.62 | 338 (17) | 163.5 (18) | 1.15E-02 |
| **hsa.miR.185.5p^b^** | 4.99 | 295 (23) | 107.5 (12) | 1.20E-02 |
| **cfa.miR.221^b^** | 2.93 | 316.5 (24) | 92 (11) | 1.25E-02 |
| **bta.miR.20b^b^** | 4.35 | 338 (23) | 133.5 (12) | 1.76E-02 |
| **hsa.miR.451a^b^** | -5.86 | 541 (11) | 145.5 (24) | 1.84E-02 |
| **hsa.miR.133b** | 3.74 | 295 (17) | 192.5 (18) | 1.96E-02 |
| **hsa.miR.22.3p^c^** | 3.027 | 316.5 (24) | 144 (11) | 2.62E-02 |
| **hsa.miR.92a.3p** | -2.50 | 268.5 (24) | 180 (11) | 2.81E-02 |
| **hsa.miR.143.3p** | 6.33 | 145.5 (22) | 383 (13) | 3.07E-02 |
| **cfa.miR.1** | 7.07 | 281.5 (26) | 144 (9) | 3.49E-02 |
| **hsa.miR.222.3p** | 2.60 | 589 (8) | 238 (27) | 3.55E-02 |
| **hsa.miR.28.3p^c^** | 3.82 | 144 (9) | 281.5 (26) | 4.09E-02 |
| **hsa.miR.16.5p** | -4.20 | 541 (11) | 163.5 (24) | 4.70E-02 |
| **cfa.miR.133c** | 6.41 | 277 (27) | 145.5 (8) | 4.94E-02 |
| **OVC2** | | | | |
| **miRNA** | **Cut-off value*** | **Median days in high group (sample #)** | **Median days in low group (sample #)** | **p-value** |
| **dme.miR.133.3p** | 4.39 | 109 (3) | 349.5 (10) | 1.16E-04 |
| **hsa.miR.28.3p** | 4.20 | 413.5 (6) | 112 (7) | 1.74E-03 |
| **hsa.miR.93.5p^b^** | 2.72 | 389 (7) | 110.5 (6) | 3.76E-03 |
| **hsa.miR.451a^b^** | -2.81 | 389 (7) | 110.5 (6) | 3.76E-03 |
| **cfa.miR.1271** | 7.59 | 349.5 (10) | 112 (3) | 6.08E-03 |
| **hsa.miR.185.5p^b^** | 6.69 | 349.5 (10) | 112 (3) | 6.08E-03 |
| **hsa.miR.151a.5p** | 3.85 | 389 (7) | 110.5 (6) | 6.92E-03 |
| **hsa.miR.19a.3p** | 1.08 | 620.5 (4) | 118 (9) | 7.09E-03 |
| **bta.miR.26b** | 2.32 | 355 (9) | 110.5 (4) | 8.77E-03 |
| **bta.miR.20b^b^** | 6.13 | 389 (7) | 121 (6) | 1.02E-02 |
| **cfa.miR.221^b^** | 6.39 | 372 (8) | 118 (5) | 1.52E-02 |
| **hsa.miR.22.3p** | 5.25 | 118 (9) | 620.5 (4) | 1.78E-02 |
| **hsa.miR.222.3p** | 3.49 | 88.5 (2) | 344 (11) | 1.90E-02 |
| **cfa.miR.23a** | 0.56 | 372 (8) | 118 (5) | 2.50E-02 |
| **gga.miR.18a.5p^b^** | 6.39 | 355 (9) | 115 (4) | 3.02E-02 |
| **hsa.miR.214.3p^a^** | 4.27 | 112 (5) | 349.5 (8) | 3.41E-02 |
| **CCOGC** | | | | |
| **miRNA** | **Cut-off value*** | **Median days in high group (sample #)** | **Median days in low group (sample #)** | **p-value** |
| **hsa.miR.92b.3p** | 4.87 | 95 (2) | 754 (11) | 1.05E-04 |
| **hsa.let.7c.5p** | 0.91 | 114 (3) | 770 (10) | 1.12E-02 |
| **hsa.miR.28.3p^c^** | 3.48 | 114 (3) | 770 (10) | 1.12E-02 |
| **dme.miR.133.3p** | 3.26 | 799 (7) | 117.5 (6) | 1.23E-02 |
| **hsa.miR.7.5p** | 2.76 | 130.5 (4) | 786 (9) | 1.57E-02 |
| **hsa.miR.214.3p^a^** | 2.27 | 117.5 (4) | 786 (9) | 1.60E-02 |
| **hsa.miR.143.3p** | 3.14 | 1198 (2) | 468 (11) | 2.36E-02 |
| **hsa.miR.885.5p** | 4.42 | 1198 (2) | 468 (11) | 2.36E-02 |
| **hsa.miR.22.3p^c^** | 1.74 | 819 (6) | 147 (7) | 3.57E-02 |

^a^ miRNAs with the same disease-free interval groups in all three populations.

^b^ miRNAs with the same disease-free interval groups in OVC1 and OVC2.

^c^ miRNAs with the same disease-free interval groups in OVC1 and CCOGC.

*Cut-off value represents the normalized Ct value of the respective miRNA which best separates the high group and low group.

Note: No miRNA predicted disease-free interval similarly between OVC2 and CCOGC.
