# Supplementary material for: Plasma microRNA signatures predict prognosis in canine osteosarcoma patients
Source: PLoS One. 2024 Dec 31;19(12):e0311104. doi: 10.1371/journal.pone.0311104 (PMC11687810; doi:10.1371/journal.pone.0311104)
Supplement: S9 Table — (DOCX) [file pone.0311104.s009.docx]

**S9 Table. MiRNAs associated with 1-year survival in each population.**

| **OVC1** | | | | |
| --- | --- | --- | --- | --- |
| **miRNA** | **Cut-off value*** | **Median days in high group (sample #)** | **Median days in low group (sample #)** | **p-value** |
| **cfa.miR.652** | 4.89 | 295 (27) | 81 (8) | 3.36E-06 |
| **hsa.miR.214.3p^c^** | 3.50 | 92 (15) | 446 (20) | 1.79E-05 |
| **gga.miR.18a.5p^b^** | 4.78 | 295 (27) | 89 (8) | 7.14E-05 |
| **hsa.miR.93.5p^b^** | 0.57 | 338 (25) | 107.5 (10) | 4.06E-04 |
| **hsa.miR.20a.5p** | 0.23 | 338 (23) | 107.5 (12) | 1.62E-03 |
| **cfa.miR.221^b^** | 2.93 | 316.5 (24) | 92 (11) | 2.15E-03 |
| **hsa.miR.378a.3p** | 4.24 | 138 (25) | 690.5 (10) | 2.22E-03 |
| **bta.miR.20b^b^** | 4.35 | 338 (23) | 133.5 (12) | 2.56E-03 |
| **hsa.miR.185.5p^b^** | 5.05 | 295 (25) | 107.5 (10) | 3.42E-03 |
| **cfa.miR.23a^c^** | -0.39 | 130.5 (14) | 338 (21) | 3.97E-03 |
| **hsa.miR.22.3p** | 3.03 | 316.5 (24) | 144 (11) | 6.52E-03 |
| **hsa.miR.19a.3p^b^** | 0.40 | 316.5 (24) | 92 (11) | 8.32E-03 |
| **hsa.miR.205.5p** | 4.32 | 360.5 (20) | 144 (15) | 8.34E-03 |
| **hsa.miR.451a^b^** | -5.86 | 541 (11) | 145.5 (24) | 8.38E-03 |
| **hsa.miR.148b.3p** | 5.94 | 286 (27) | 115 (8) | 1.01E-02 |
| **hsa.miR.16.5p** | -4.20 | 541 (11) | 163.5 (24) | 2.13E-02 |
| **hsa.miR.145.5p** | 1.34 | 162 (26) | 438 (9) | 2.27E-02 |
| **cfa.miR.142** | 1.82 | 281.5 (28) | 86 (7) | 2.38E-02 |
| **hsa.miR.126.5p** | 3.60 | 133.5 (14) | 286 (21) | 2.46E-02 |
| **hsa.miR.222.3p** | 4.43 | 281.5 (26) | 92 (9) | 3.59E-02 |
| **hsa.miR.128.3p** | 3.02 | 541 (9) | 145.5 (26) | 3.71E-02 |
| **cfa.miR.1271** | 5.61 | 138 (13) | 290.5 (22) | 4.34E-02 |
| **OVC2** | | | | |
| **miRNA** | **Cut-off value*** | **Median days in high group (sample #)** | **Median days in low group (sample #)** | **p-value** |
| **dme.miR.133.3p** | 4.39 | 109 (3) | 349.5 (10) | 1.16E-04 |
| **hsa.miR.19a.3p^b^** | 1.44 | 360 (8) | 112 (5) | 8.79E-04 |
| **hsa.miR.28.3p** | 4.20 | 365 (6) | 112 (7) | 1.74E-03 |
| **hsa.miR.93.5p^b^** | 2.72 | 365 (7) | 110.5 (6) | 3.76E-03 |
| **hsa.miR.451a^b^** | -2.81 | 365 (7) | 110.5 (6) | 3.76E-03 |
| **cfa.miR.1271** | 7.59 | 349.5 (10) | 112 (3) | 6.08E-03 |
| **hsa.miR.185.5p^b^** | 6.69 | 349.5 (10) | 112 (3) | 6.08E-03 |
| **hsa.miR.151a.5p** | 3.85 | 365 (7) | 110.5 (6) | 6.92E-03 |
| **bta.miR.26b** | 2.32 | 355 (9) | 110.5 (4) | 8.77E-03 |
| **bta.miR.20b^b^** | 6.13 | 365 (7) | 121 (6) | 1.02E-02 |
| **cfa.miR.221^b^** | 6.39 | 360 (8) | 118 (5) | 1.52E-02 |
| **gga.miR.18a.5p^b^** | 6.39 | 355 (9) | 115 (4) | 3.02E-02 |
| **CCOGC** | | | | |
| **miRNA** | **Cut-off value*** | **Median days in high group (sample #)** | **Median days in low group (sample #)** | **p-value** |
| **hsa.miR.92b.3p** | 4.87 | 95 (2) | 365 (11) | 1.05E-04 |
| **cfa.miR.144** | 0.64 | 112 (2) | 365 (11) | 7.39E-03 |
| **hsa.miR.214.3p^c^** | 0.35 | 100.5 (2) | 365 (11) | 1.07E-02 |
| **hsa.let.7c.5p** | 0.91 | 114 (3) | 365 (10) | 1.12E-02 |
| **hsa.miR.28.3p** | 3.48 | 114 (3) | 365 (10) | 1.12E-02 |
| **hsa.miR.125b.5p** | 1.61 | 121 (7) | 365 (6) | 1.16E-02 |
| **dme.miR.133.3p** | 3.26 | 365 (7) | 117.5 (6) | 3.35E-02 |
| **hsa.miR.143.3p** | 4.41 | 134 (8) | 365 (5) | 3.67E-02 |
| **cfa.miR.23a^c^** | -0.11 | 134 (8) | 365 (5) | 3.67E-02 |
| **cfa.miR.125a** | 2.03 | 117.5 (4) | 365 (9) | 4.48E-02 |

^b^ miRNAs with the same disease-free interval groups in OVC1 and OVC2.

^c^ miRNAs with the same disease-free interval groups in OVC1 and CCOGC.

*Cut-off value represents the normalized Ct value of the respective miRNA which best separates the high group and low group.

Note: No miRNA predicted disease-free interval similarly between OVC2 and CCOGC.
