# Supplementary material for: Plasma microRNA signatures predict prognosis in canine osteosarcoma patients
Source: PLoS One. 2024 Dec 31;19(12):e0311104. doi: 10.1371/journal.pone.0311104 (PMC11687810; doi:10.1371/journal.pone.0311104)
Supplement: S10 Table — (DOCX) [file pone.0311104.s010.docx]

**S10 Table. Univariate analyses of clinical characteristics to outcome parameters.**

|  | **Overall survival** | | **Disease-free interval** | | **1-year survival** | |
| --- | --- | --- | --- | --- | --- | --- |
| **Variable** | **HR (95% CI for HR)** | **p-value** | **HR (95% CI for HR)** | **p-value** | **HR (95% CI for HR)** | **p-value** |
| **Age** | 1.1 (0.92-1.3) | 0.31 | 1.1 (0.9-1.3) | 0.42 | 1.1 (0.9-1.3) | 0.5 |
|  | | | | | | |
| **Sex** | | | | | | |
| **Male castrated** | 1.2 (0.64-2.4) | 0.52 | 1.3 (0.59-2.7) | 0.54 | 1.3 (0.7-2.6) | 0.38 |
| **Male intact** | 3.7 (0.48-29) | 0.21 | 3.7 (0.48-29) | 0.21 | 2.3 (0.3-17) | 0.42 |
| **Female spayed** | 0.76 (0.39-1.5) | 0.41 | 0.73 (0.34-1.6) | 0.42 | 0.72 (0.37-1.4) | 0.34 |
| **Female intact** | 0.85 (0.12-6.3) | 0.88 | 0.85 (0.12-6.3) | 0.88 | 0.76 (0.1-5.6) | 0.79 |
|  | | | | | | |
| **Weight** | 1 (0.97-1) | 0.98 | 0.99 (0.96-1) | 0.70 | 1 (0.97-1) | 0.88 |
|  | | | | | | |
| **ALP status at diagnosis** | | | | | | |
| **High** | 2 (0.6-6.8) | 0.26 | 2 (0.6-6.8) | 0.26 | 1.5 (0.43-4.9) | 0.54 |
| **Normal** | 0.76 (0.3-2) | 0.58 | 0.74 (0.25-2.2) | 0.58 | 0.85 (0.33-2.2) | 0.74 |
| **Low** | 0.84 (0.2-3.5) | 0.81 | 0.66 (0.09-4.9) | 0.68 | 0.9 (0.22-3.8) | 0.89 |
|  | | | | | | |
| **Location*** | | | | | | |
| **Femur** | 0.79 (0.28-2.2) | 0.66 | 0.75 (0.23-2.5) | 0.64 | 0.73 (0.26-2.1) | 0.55 |
| **Humerus** | 1.9 (0.85-4) | 0.12 | **2.8 (1.2-6.3)** | **0.015** | 1.9 (0.86-4) | 0.12 |
| **Radius** | 0.55 (0.27-1.1) | 0.10 | **0.3 (0.11-0.78)** | **0.014** | 0.56 (0.28-1.1) | 0.11 |
| **Tibia** | 1.2 (0.61-2.5) | 0.55 | 1.4 (0.63-3.1) | 0.41 | 1.3 (0.64-2.7) | 0.46 |

Note: Hazard ratio (HR); confidence interval (CI). Statistically significant HRs are bolded with their associated p-values

*Only bone and not location within bone was used for analysis
