# Supplementary material for: Plasma microRNA signatures predict prognosis in canine osteosarcoma patients
Source: PLoS One. 2024 Dec 31;19(12):e0311104. doi: 10.1371/journal.pone.0311104 (PMC11687810; doi:10.1371/journal.pone.0311104)
Supplement: S1 Fig — (DOCX) [file pone.0311104.s011.docx]

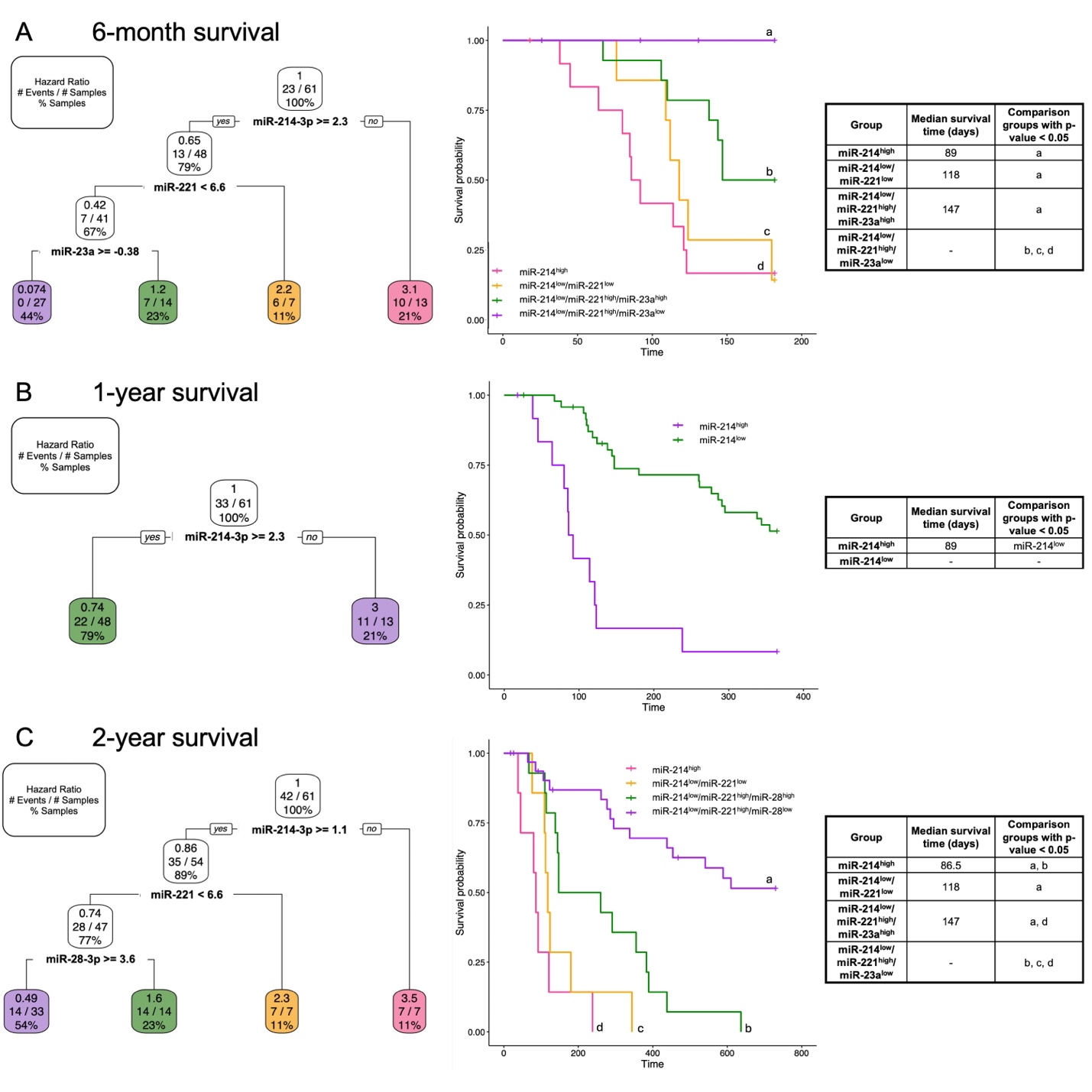


**S1 Fig. Multiple miRNA models for 6-month, 1-year, and 2-year survival.** Pruned decision tree. Each step includes the corresponding hazard ratio, number of events defined by death due to osteosarcoma prior to the time of interest out of the total number of samples, and percentage samples in each group. Kaplan-Meier survival curve for each of the groups. Median survival time (days) for each group and corresponding p-value between group comparisons. (A) 6-month survival. (B) 1-year survival. (C) 2-year survival.
