# Supplementary material for: Plasma microRNA signatures predict prognosis in canine osteosarcoma patients
Source: PLoS One. 2024 Dec 31;19(12):e0311104. doi: 10.1371/journal.pone.0311104 (PMC11687810; doi:10.1371/journal.pone.0311104)
Supplement: Attachment — Submitted filename: Ludwig Response to reviewers.R1.docx [file pone.0311104.s012.docx]

Journal requirements:

When submitting your revision, we need you to address these additional requirements.

1. Please ensure that your manuscript meets PLOS ONE's style requirements, including those for file naming. The PLOS ONE style templates can be found at

<https://journals.plos.org/plosone/s/file?id=wjVg/PLOSOne_formatting_sample_main_body.pdf> and

<https://journals.plos.org/plosone/s/file?id=ba62/PLOSOne_formatting_sample_title_authors_affiliations.pdf>

These modifications have been made to fit the examples provided in the links above. Please provide specific notes if further modifications are required.

2. We note that you have included the phrase “data not shown” in your manuscript. Unfortunately, this does not meet our data sharing requirements. PLOS does not permit references to inaccessible data. We require that authors provide all relevant data within the paper, Supporting Information files, or in an acceptable, public repository. Please add a citation to support this phrase or upload the data that corresponds with these findings to a stable repository (such as Figshare or Dryad) and provide and URLs, DOIs, or accession numbers that may be used to access these data. Or, if the data are not a core part of the research being presented in your study, we ask that you remove the phrase that refers to these data.

This statement has been removed from lines 265 as it is not a core part of the research being presented.

3. We notice that your supplementary tables are included in the manuscript file. Please remove them and upload them with the file type 'Supporting Information'. Please ensure that each Supporting Information file has a legend listed in the manuscript after the references list.

This has been updated the Supporting Information files separated from the main manuscript.

[Note: HTML markup is below. Please do not edit.]

Reviewers' comments:

Reviewer's Responses to Questions

**Comments to the Author**

1. Is the manuscript technically sound, and do the data support the conclusions?

The manuscript must describe a technically sound piece of scientific research with data that supports the conclusions. Experiments must have been conducted rigorously, with appropriate controls, replication, and sample sizes. The conclusions must be drawn appropriately based on the data presented.

Reviewer #1: Yes

Reviewer #2: Yes

Reviewer #3: Yes

2. Has the statistical analysis been performed appropriately and rigorously?

Reviewer #1: No

Reviewer #2: Yes

Reviewer #3: Yes

3. Have the authors made all data underlying the findings in their manuscript fully available?

The [PLOS Data policy](http://www.plosone.org/static/policies.action#sharing) requires authors to make all data underlying the findings described in their manuscript fully available without restriction, with rare exception (please refer to the Data Availability Statement in the manuscript PDF file). The data should be provided as part of the manuscript or its supporting information, or deposited to a public repository. For example, in addition to summary statistics, the data points behind means, medians and variance measures should be available. If there are restrictions on publicly sharing data—e.g. participant privacy or use of data from a third party—those must be specified.

Reviewer #1: No

Reviewer #2: Yes

Reviewer #3: Yes

4. Is the manuscript presented in an intelligible fashion and written in standard English?

PLOS ONE does not copyedit accepted manuscripts, so the language in submitted articles must be clear, correct, and unambiguous. Any typographical or grammatical errors should be corrected at revision, so please note any specific errors here.

Reviewer #1: Yes

Reviewer #2: Yes

Reviewer #3: Yes

5. Review Comments to the Author

Please use the space provided to explain your answers to the questions above. You may also include additional comments for the author, including concerns about dual publication, research ethics, or publication ethics. (Please upload your review as an attachment if it exceeds 20,000 characters)

Reviewer #1: The authors performed a clinical search of microRNA biomarkers in plasma that could be predictive of OSA diagnosis and prognosis in domestic dogs.

Since my expertise is mostly computational and statistical, although I hold a clinical background, I will focus my review in these aspects throughout the paper, leaving additional minor details for further reviews, in case they might happen in successive rounds. I leave other possibly major issues up to the consideration of additional reviewers with more expertise in the fields I will not touch upon here. Below are my comments:

1.In Table S1, the authors report to have used a panel of 56 probes for their analyses using prior information and a separate trial for expression detection for which there are no shown results in the manuscript, as said in Methods in the “Target miRNA selection” section.

The QIAGEN canine miScript miRNome is a commercially available array containing 277 miRNAs found to be widely expressed by their internal investigation. They have been validated by QIAGEN to match the mature canine sequence. Interested readers can find the panel details on QIAGEN’s website and we have referenced our previous PLOS ONE paper (Craig et al, 2019) that used this product.

Additional information about the miRNA primers used, such as the forward and reverse sequence could be a good addition to the supplementary material, adding clarity to the data exposition.

For the custom panels, QIAGEN’s commercially available validated probes were used. A sentence providing further information on where these sequences can be found has been added to line 222-224.

2.Then, in Table S4, they report a subset of these 56 miRNAs for the differential expression analyses using RT-qPCR data (44). I would like to be able to check the original Ct values and successive normalization steps up until reaching the reported Fold Changes in Table S4 and Table 1. Could the authors provide that? In this way, it could be possible to get access to the “expression” estimates of each miRNA before FC and significance calculation, and therefore better assess the real effect size that a given FC represents. Since high or low FC can greatly vary in their interpretation given the expression levels of the miRNAs in question between groups, adding such information would provide a better source of analysis of the results shown in the manuscript.

Calibrated Ct values of all samples included in the analyses described within the manuscript have been included as a supplemental file.

3.The authors used a set of microRNAs that include both canine (cfa-) and non-canine (hsa-, bta-, rno-...) probes, which I assume correspond to orthologs of existing expressed canine miRNAs. However, inferring this from the results presented is not straightforward. Could the authors include a supplementary table with the sequence of the mature miRNAs targeted by qPCR, both comparing the canine and non-canine orthologs? To my understanding, these non-canine miRNA primers should target sequences that are conserved among the two species, at least, the domestic dog and the species from which the miRNA primer is derived. It would be really helpful as a cross-check to be able to see that the non-canine miRNAs used are actually equivalent to real existing and expressed canine mature miRNAs circulating in plasma.

QIAGEN’s commercially available probes were used which have been validated in their respective species and shown to have cross-species sequence homology. A sentence providing further information on where these sequences can be found has been added to line 222-224. It is publicly available on QIAGEN’s GeneGlobe website and we have provided the catalog numbers for ease of access to this information. We think that including the publicly available sequence information again in the manuscript is not needed.

4.Presenting the significance of the differential expression the way the authors did could be misleading. First, it would help the reader to get access, both in the main and in the supplementary tables, to the accurate p-value number estimated for each miRNA in each contrast. Simplifying p-values to ranges can obscure differences in significance among different contrasts. A scientific notation of the p-values could also help for a better interpretation.

We agree, and have changed the p-values that represented ranges (such as < 0.05 etc.) to their respective scientific notation in the tables as requested. We have left these as ranges in the figures for readability.

Also, the authors should try to correct their p-values with a multiple testing approach, such as FDR or similar, so as to see whether their estimated p-values survive multiple testing correction thresholding. Maybe the reported cfa-miR-214-3p keeps being the only significant one after FDR correction.

Thank you for this suggestion. We have now applied a multiple testing correction. We used the Benjamini-Hochberg procedure as described in line 311-312.
The corrected p-values have now been reported in Table 1. We only reported the corrected values in this Table due to limitations in size of the columns, but the original and corrected p-values are available for all miRNAs in Supplemental Table S5, which has been altered to include these values along with the original p-values.

5.Subsetting differential expression analysis tables the way the authors did in the Table 1 could be misleading. I recommend to include all p-values, either significant or not, in scientific notation, removing ranges and providing the real estimate number and adding a column with the multiple testing corrected value (via FDR or similar).
As we had numerous results with differential expression, only those with statistical significance were included and highlighted to demonstrate those in which there is confidence in a true differential expression. All data is available, however we strongly wish to keep the results as presented. As described above, we have changed the significant p-values to scientific notation as requested and added a column with the multiple testing corrected values.

Given these points, I recommend Major revision of the manuscript.

Reviewer #2: Following recommendations are suggested:
1) kindly format the paragraph alignment. Done.
2) arrange the manuscript sections as per journal guidelines. Done.
3) provide the Supplementary data in separate file. These files have been separated and modified

4)it is recommended to include S2,S3,S4 in manuscript, not as supplementary data because this information needed a spotlight.

We have summarized this data within the manuscript and have included additional details that we felt to be beyond the scope of the main manuscript in the supplemental tables for interested readers. We feel that including this data in the main paper would negatively impact the clarity of the main findings for the general audience of PLOS One.

5) how did you calculate p-value?. Which statistical approach or model is used for statistical analysis?.

For the delta Ct comparison a Wilcoxon-rank sum test was performed (line 291); All Kaplan-Meier survival curves used a log-rank test (lines 306, 343); A Mann-Whitney U test was used for hemolysis assessment (line 258); Cox regression was used for univariate analyses (line 334); A Bonferroni multiple comparisons correction was used for the pruned decision tree models (line 344); one-way ANOVA was used to compare clinical outcome parameters (line 374).

6) which significance levels are used as reference?. Kindly mention.

All tests were considered statistically significant if p < 0.05 as mentioned in the materials and methods section. (Lines 292, 306, 331, 344)

7) kindly arrange table 1 at end of manuscript text,before figures.

The submission guidelines state to include the table directly after the paragraph in which it is mentioned.

Reviewer #3: The paper is well written and explores the use of plasma miRNAs to predict outcomes in dogs with osteosarcoma. It would be useful to include information regarding treatment of the dogs since this may impact their disease progression and survival. Although possibly beyond the scope of this paper, it could be also be informative to discuss the putative functions of the miRNAs that were selected as predictors in the pruned tree models.

We have included information regarding treatment status in that all patients received amputation, along with at least one dose of cytotoxic chemotherapy (line 137). While all dogs included met this criteria, any differences in treatment were not analyzed. Some dogs died very early in the course of treatment. Excluding dogs that did not complete all their chemotherapy rounds would bias the study towards longer surviving cases, which would remove a significant proportion of patients.

6. PLOS authors have the option to publish the peer review history of their article ([what does this mean?](https://journals.plos.org/plosone/s/editorial-and-peer-review-process#loc-peer-review-history)). If published, this will include your full peer review and any attached files.


If you choose “no”, your identity will remain anonymous but your review may still be made public.


**Do you want your identity to be public for this peer review?** For information about this choice, including consent withdrawal, please see our [Privacy Policy](https://www.plos.org/privacy-policy).

Reviewer #1: No

Reviewer #2: **Yes:** Syeda Zahra Abbas

Reviewer #3: No
