# Supplementary material for: Plasma microRNA signatures predict prognosis in canine osteosarcoma patients
Source: PLoS One. 2024 Dec 31;19(12):e0311104. doi: 10.1371/journal.pone.0311104 (PMC11687810; doi:10.1371/journal.pone.0311104)
Supplement: Attachment — Submitted filename: Response to reviewers.R2.docx [file pone.0311104.s013.docx]

Journal Requirements:

Please review your reference list to ensure that it is complete and correct. If you have cited papers that have been retracted, please include the rationale for doing so in the manuscript text, or remove these references and replace them with relevant current references. Any changes to the reference list should be mentioned in the rebuttal letter that accompanies your revised manuscript. If you need to cite a retracted article, indicate the article’s retracted status in the References list and also include a citation and full reference for the retraction notice.

The reference list has been checked for completeness. Two DOIs have been corrected. To our knowledge none of the cited papers have been retracted.

Additional Editor Comments:

Reviewer #1:

Dear Authors,

You have mostly addressed all my previous comments and thereby I will only add three minor additions to be considered for a better clarity and accessibility of your work. In my opinion, they should be implemented before the manuscript is considered acceptable for publication:

1. In line 192, could you please provide a link to the Qiagen miRNA catalog you used that actually directs you to the said catalog? As it is right now it only leads to a generic webpage of the company, which adds no relevant info to the manuscript.

To access the catalog, you are required to have a free account and log into their system to design a plate to review all of this information. We have now provided the most direct link (line 224); however, this still requires login. This information is freely and publicly available in this fashion and therefore does not benefit being provided again in the manuscript as the probe name and Catalog No. are provided for those interested in replicating or referencing our findings.

2. I would suggest that authors include Supplementary Tables as Excel tables, as they have done with Table S10, instead of using docx plain files. In this way, it would be easier to get access to the info contained in them.

We have elected to maintain the tables in a Word format for these existing supplementary tables as they are not as large as Table S10.

3. You have added FDR-corrected Pvalues to Table 1, which seems to be a subset of Table S4. In the answer to my initial request no 5 where I asked the FDR values to be included in the main table and the Supplementary Tables, the authors refer that this info has been added to Table S5. I guess they actually meant Table S4, which is the one that includes differential expression analyses results. However, the current Table S4 only includes P-values, and not FDR values, as far as I can see. For this table to be correct and matchin Table 1, you should add the corresponding Pvalues AND the FDR values (both in scientific notation) to all the miRNAs included, which then would lead to the subset shown in the main table in the manuscript.

Table S5 is the corresponding table to Table 1 in the manuscript. The FDR-corrected values have been added alongside the p-values as requested. These changes have not been tracked as it was extensive re-formatting.

Given these coments, I consider the manuscript needs Minor Revision.
